# MAFsnp: A Multi-Sample Accurate and Flexible SNP Caller Using Next-Generation Sequencing Data

**DOI:** 10.1371/journal.pone.0135332

**Published:** 2015-08-26

**Authors:** Jiyuan Hu, Tengfei Li, Zidi Xiu, Hong Zhang

**Affiliations:** 1 State Key Laboratory of Genetic Engineering and Institute of Biostatistics, School of Life Sciences, Fudan University, 220 Handan Road, Shanghai 200433, P. R. China; 2 HKUST Jockey Club Institute for Advanced Study, The Hong Kong University of Science and Technology, Hong Kong, P.R. China; Chinese Academy of Science, CHINA

## Abstract

Most existing statistical methods developed for calling single nucleotide polymorphisms (SNPs) using next-generation sequencing (NGS) data are based on Bayesian frameworks, and there does not exist any SNP caller that produces p-values for calling SNPs in a frequentist framework. To fill in this gap, we develop a new method MAFsnp, a Multiple-sample based Accurate and Flexible algorithm for calling SNPs with NGS data. MAFsnp is based on an estimated likelihood ratio test (eLRT) statistic. In practical situation, the involved parameter is very close to the boundary of the parametric space, so the standard large sample property is not suitable to evaluate the finite-sample distribution of the eLRT statistic. Observing that the distribution of the test statistic is a mixture of zero and a continuous part, we propose to model the test statistic with a novel two-parameter mixture distribution. Once the parameters in the mixture distribution are estimated, p-values can be easily calculated for detecting SNPs, and the multiple-testing corrected p-values can be used to control false discovery rate (FDR) at any pre-specified level. With simulated data, MAFsnp is shown to have much better control of FDR than the existing SNP callers. Through the application to two real datasets, MAFsnp is also shown to outperform the existing SNP callers in terms of calling accuracy. An R package “MAFsnp” implementing the new SNP caller is freely available at http://homepage.fudan.edu.cn/zhangh/softwares/.

## Introduction

The development of next-generation sequencing (NGS) technologies in the past few years has transformed today’s biological science [1]. With cheap and ultra-high throughput characteristics [2], the NGS technologies have been widely applied to a vast number of biological branches [3–7]. Many projects such as the 1000 Genomes Project [8, 9], the Cancer Genome Atlas Project [10], the NHLBI Exome Sequencing Project [11] have been carried out, trying to elucidating all forms of human hereditary polymorphism. Single-nucleotide polymorphisms (SNPs) are commonly seen in many conceivable biological processes such as microRNA binding site [12], transcriptional regulation [13], protein coding [14], and so on. Detecting SNPs has been considered as an essential step in NGS data analysis.

A variety of SNP calling tools have been developed to identify SNPs using single or multiple sample(s) [15–22]. Based on a Bayesian rule, MAQ [15] assumes that there are at most two alleles at a locus and reports posterior probabilities of three possible genotypes for each individial. A SNP is called if a heterozygous genotype or homozygous variant genotype is reported. SOAPsnp [16] is based on another Bayesian model for ten possible genotypes, and prior information such as dbSNP [23] can be integrated into this SNP caller. VarScan [17] calls SNPs with several filters on coverage, quality, variant frequency, and strand-specific depths. MAQ, SOAPsnp, and VarScan are applicable to a single sample or pooled samples, but it is difficult for them to efficiently utilize the NGS data of multiple samples to call SNPs. An increasing number of tools have been developed to call SNPs using NGS data from multiple samples. seqEM [18] is a genotype caller utilizing multi-sample NGS data in a Bayesian framework, which can also be used to call SNPs. QCALL [19] effectively utilizes linkage disequilibrium information to call SNPs with multi-sample low coverage data. Atlas-SNP2 [20] adopts a Bayesian approach for multiple samples to call SNPs based on a logistic regression model for mapping/sequencing error. To call SNPs using either single-sample or multi-sample NGS data, both GATK [21] and SAMtools [22] use a Bayesian rule to infer the posterior probability of being a SNP followed by a tedious filtering step.

In each of the above SNP callers, either quality score or posterior probability is provided as a measure of confidence of being a SNP, and some threshold of the quality score or posterior probability is suggested for calling SNPs. Unfortunately, it is hard for users to determine their own threshold in these SNP callers to control required false discovery rate (FDR). It is thus desired to have a statistical method providing a p-value (a commonly used measure of significance measure for general hypothesis testing) for each candidate locus, which can be easily used to control FDR. MAFsnp, a Multi-sample Accurate and Flexible SNP caller, is designed in this article for this purpose.

MAFsnp is based on a likelihood function for the NGS read counts from multiple samples, and the SNP calling issue is transformed into a hypothesis testing problem on the minor allele frequency (MAF) for each candidate locus, then an estimated likelihood ratio test (eLRT) statistic is used to detect SNPs. The null distribution of the eLRT statistic is essential in calculating a p-value. Note that the eLRT statistic is a function of mapping/sequencing error rate and minor allele frequency. Because the mapping/sequencing error rate is usually very small and it is close to the boundary of the parameter space, the finite sample distribution of the eLRT statistic could greatly deviate from the standard limiting distribution. Through extensive simulations, we find that the test statistic is a mixture distribution of zero and a continuous distribution. We propose to approximate the distribution of the continuous part with a scaled chi-square distribution. An algorithm is then developed to estimate the scale parameter and the proportion of zero part, and p-values for detecting SNPs follows immediately. Using these p-values, multiple-testing corrected p-values can be easily obtained to control FDR. One key feature of MAFsnp is that it only uses summarized read count data. As a result, MAFsnp is applicable to the NGS data generated from any sequencing platform.

The rest of this article is organized as follows. First, a new distribution family is used to model the read count data with mapping/sequencing error, and a rigorous statistical method is developed to call SNPs based on this model. Second, a simulation study is conducted to evaluate the performance of MAFsnp and several existing SNP callers, which demonstrates that MAFsnp could have much better control of FDR than the competitors. For example, in one of our simulation situations, the FDRs of SAMtools, GATK, MAQ, seqEM, and MAFsnp were 8.7 × 10^−5^, 0.157, 0.003, 0.061, and 0.011, repectively (nominal FDR level = 0.01). Third, the application to two real datasets further verifies that MAFsnp could outperform the competitors in terms of calling accuracy. For example, based on a fragment of public sequencing data, the accuracy of MAFsnp was 91.2%, compared with 31.7%, 59.0%, 90.3%, and 88.8% by seqEM, MAQ, GATK, and SAMtools, respectively. Finally, some concluding remarks are given in Discussion.

## Methods

### Notation and model

We will transform the SNP calling issue into a hypothesis testing problem. In our method, we only consider diploid organisms, and we only use read counts mapped to a reference genome. For any nucleotide locus, denote by *R* and *r* the reference allele and variant allele, respectively. Let *p* denote the frequency of the variant allele *r* in a general population, which is called minor allele frequency (MAF) throughout this article. By definition, a locus is a SNP if and only if *p* > 0. Therefore, the SNP calling issue can be transformed into the following hypothesis testing problem:

$$H_{0}\;:\;p=0\;\text{versus}\;H_{a}\;:\;p>0.$$ (1)

Suppose that we have *J* nucleotide loci. For the *j*th locus with reference allele *R*_*j*_ and variant allele *r*_*j*_, let *p*_*j*_ denote its MAF (the population frequency of *r*_*j*_). We assume that the Hardy-Weinberg equilibrium holds, so that the population frequencies of the genotypes *R*_*j*_ *R*_*j*_, *R*_*j*_ *r*_*j*_, and *r*_*j*_ *r*_*j*_ are (1 − *p*_*j*_)^2^, 2*p*_*j*_(1 − *p*_*j*_), and $p_{j}^{2}$, respectively. Suppose that read counts are available from *n* independent samples. For the *j*th (*j* = 1, …, *J*) locus of the *i*th (*i* = 1, …, *n*) sample, let *G*_*ij*_ denote the unknown genotype, *N*_*ij*_ the total number of reads, and *X*_*ij*_ the number of variant reads. Let **N**_**j**_ = (*N*_*j*1_, ⋯, *N*_*jn*_)^*T*^ and **X**_**j**_ = (*X*_*j*1_, ⋯, *X*_*jn*_)^*T*^. Because of mapping/sequencing error, the genotype *r*_*j*_ *r*_*j*_ or *R*_*j*_ *r*_*j*_ could be observed even if the the nucleotide locus is not a SNP. As done in literature [18], we assume that the mapping/sequencing error is symmetric, i.e.,

Pr(read=R|trueallele=r)=Pr(read=r|trueallele=R).

Let *e*_*j*_ denote the common mapping/sequencing error rate at the *j* locus, and let *θ*_*j*_ = (*e*_*j*_, *p*_*j*_) denote the unknown parameter vector for the *j*th locus. It is reasonable to assume that the observed number of variant reads follows a binomial distribution given the true genotype and the total number of reads,. Therefore, we have the following log-likelihood function for the observed read counts:

$$\begin{array}{cc} l_{j}\left(\theta_{j};X_{j},N_{j}\right) & =\sum_{i=1}^{n}\log\mathrm{Pr}(X_{ij},G_{ij}|N_{ij},\theta_{j}),\\ & =\sum_{i=1}^{n}\log\{p_{j}^{2}B\left(X_{ij},N_{ij},1-e_{j}\right)+2p_{j}\left(1-p_{j}\right)B\left(X_{ij},N_{ij},0.5\right)+{\left(1-p_{j}\right)}^{2}B\left(X_{ij},N_{ij},e_{j}\right)\}, \end{array}$$ (2)

where

B(Xij,Nij,ej)=(NijXij)ejXij(1-ej)Nij-Xij

is the probability mass function of the binomial distribution with trial number *N*_*ij*_ and successful probability *e*_*j*_. The likelihood ratio test (LRT) statistic for tesing *H*_0_ : *p*_*j*_ = 0 is

$$2\;\{\max_{e_{j},p_{j}}l_{j}\left(\left(e_{j},p_{j}\right);X_{j},N_{j}\right)-\max_{e_{j}}l_{j}\left(\left(e_{j},0\right);X_{j},N_{j}\right)\}.$$ (3)

Note that the maximum lieklihood estimator (MLE) of *θ*_*j*_ under *H*_0_ has a closed form: ${\tilde{\theta }}_{j}=({\tilde{e}}_{j},0)$ with ${\tilde{e}}_{j}=\sum_{i}X_{ij}/\sum_{i}N_{ij}$. The evaluation of the LRT statistic Eq (3) involves maximizing the log-likelihood function with respect to a 2-dimensional parameter vector. We found through a preliminary simulation study that the conventional algorithms designed for finding local a maximizer/minimizer are usually slow to converge. To avoid this problem, we consider the following eLRT statistic defined by

$$T_{j}=2\;\{\max_{p_{j}}l_{j}\left(\left({\tilde{e}}_{j},p_{j}\right);X_{j},N_{j}\right)-l_{j}\left(\left({\tilde{e}}_{j},0\right);X_{j},N_{j}\right)\}.$$ (4)

Through our preliminary simulation study, we find that the power of this eLRT is very close to that of the original LRT, but the computational speed of the former is about 120 times that of the later. Therefore, we propose to use Eq (4) instead of Eq (3).

### Null distribution of the eLRT statistic

The null distribution of the eLRT statistic is essential for calculating p-values. According to the standard large sample theory, the limiting null distribution of *T*_*j*_ is a centralized chi-square distribution (mixed chi-square distribution) provided that the true parameter vector under the null hypothesis is an inner point (on the boundary) of the parameter space and some other regularity conditions hold. In our model, the parameter vector under the null hypothesis is on the boundary of the parameter space, and the limiting null distribution of *T*_*j*_ is a mixture of 0 and the centralized chi-square distribution of 1 df [24]:

$$D_{0.5,1}\;≔\;0.5\cdot 0+0.5\cdot \chi_{1}^{2}.$$ (5)

In real situation, the mapping/sequencing error rate is typically small. For example, the mapping/sequencing error rate is around 0.01 for the Illumina Hiseq 2000 platform. As a result, the finite sample null distribution of *T*_*j*_ could greatly deviate from the limiting distribution Eq (5). In some of our preliminary simulations, we find that the proportion of zero part (*T*_*j*_ = 0) could be very close to 1, and the non-zero part of *T*_*j*_ could also deviate from the chi-square distribution $\chi_{1}^{2}$. Motivated by this observation, we propose to approximate the null distribution of *T*_*j*_ by the following modified mixture distribution:

$$D_{a,k}\;≔\;a\cdot 0+\left(1-a\right)\cdot \left(k\chi_{1}^{2}\right),$$ (6)

where $k\chi_{1}^{2}$ is a scaled chi-square distribution with expectation *k*. Obviously, *D*_0.5,1_ is a special case of *D*_*a*,*k*_. The p-value based on the distribution *D*_*a*,*k*_ is $\left(1-a)\chi_{1}^{2}(T_{j}/k\right)$, where $\chi_{1}^{2}(x)$ is the upper *x*-quantile of the chi-square distribution of 1 df. For convenience, hereafter, the methods based on *D*_0.5,1_ and *D*_*a*,*k*_ are called MAFsnp0 and MAFsnp, respectively.

We can theoretically show that *a* could be much bigger than 0.5 under some simple conditions. For example, with a constant coverage of *N* = 2, a mapping/sequencing error rate of *e* = 0.01, and a sample size of *n* = 100, the proportion of zero part of *T*_*j*_ (i.e., *a*) is approximatly 0.99. Refer to Theorems 1 and 2 in S1 Method for details. In the general situation, we can estimate *a* and *k* using genewise information. To this end, we first identify *J* null loci that are not SNPs. In practice, some SNP information can be obtained from a public database like dbSNP [23], and the remaining loci can be treated as null loci since most of them should not be SNPs. Let *J*_0_ be the number of null loci with *T*_*j*_ = 0, then we can estimate *a* by

$$\hat{a}=\frac{J_{0}}{J}.$$

Let the positive values of test statistics for the other *J* − *J*_0_ null loci be ${\tilde{T}}_{s}$ (*s* = 1, …, *J* − *J*_0_), then we can estimate *k* with

$$\hat{k}=\frac{1}{\sum_{s=1}^{J-J_{0}}1_{\left\{T_{s}\leq 15\right\}}}\sum_{s=1}^{J-J_{0}}{\tilde{T}}_{s}1_{\left\{T_{s}\leq 15\right\}},$$

where 1_{*T*_*s*_ ≤ 15}_ = 1 if *T*_*s*_ ≤ 15 and 0 othewise. Here we use a trimmed sample mean instead of the ordinary sample mean. This can effectively reduce the impact of large outliers. The threshold 15 is around the upper 10^−4^ quantile of the chi-square distribution of 1 df, so that very few non-outliers would be excluded for calculating $\hat{k}$.

### Comparison metrics

In our simulation studies, we will compare the performance of the considered SNP callers through FDR. Here *FDR* represents the fraction of non-SNPs in the positive SNP list. Given a list of p-values for detecting SNPs, FDR corrected p-values can be obtained using the R function “p.adjust” for the purpose of controlling FDR. A good SNP caller should have its FDR controlled around the nominal level. In literature, *precision* is defined as 1 minus FDR. Among all SNP callers with the same precision, the one with highest *recall* (true positive rate) would be preferred.

It is impossible to have a SNP caller with both precision and recall uniformly larger than other SNP callers. The following *F*_1_ score is commonly used to balance precision and recall:

$$F_{1}=2\cdot \frac{\text{precision}\;\times \;\text{recall}}{\text{precision}+\text{recall}}.$$ (7)

It is seen that 0 ≤ *F*_1_ ≤ 1, and the *F*_1_ score serves as a measure of accuracy, that is, a larger value of *F*_1_ score suggests a higher accuracy.

For simulated data, precision and recall at any given threshold of p-values can be calculated because the SNP information is known in advance. As a result, a precision-recall curve can be drawn for any SNP caller provided p-values are available. Hereafter, the maximal *F*_1_ score on the recision-recall curve is termed *F*_*max*_. For any real dataset, the true SNPs might be unknown, but we can use the SNP information in the dbSNP as a reference, and precision and recall can be correspondingly estimated.

Transition/transversion (Ti/Tv) ratio is shown to be another useful index indicating false positive rate (FPR) in practice [21]. *Transitions* are interchanges of two-ring purines base (*A* ↔ *G*) or one-ring pyrimidines (*C* ↔ *T*), while *transversions* are interchanges of purine for pyrimidine bases. Recent human studies show that the Ti/Tv ratio for whole human genome is around 2.0∼2.1 [9]. Generally, a higher Ti/Tv ratio implies a lower FPR.

## Data simulation

The inputs of the SNP callers seqEM and MAFsnp are counts of the reads mapped to a reference, while SAMtools, GATK, and MAQ only accept BAM/SAM/cns files containing mapped sequence reads. Note that seqEM and MAFsnp also accept mapped sequence reads since the reads can be counted for each nucleotide locus. Accordingly, we considered generating both read count data and sequence data. The counts data were generated from binomial distributions, while the sequence data were generated using the module *wgsim* in SAMtools.

### Read count data

In order to assess the performance of MAFsnp, MAFsnp0, and seqEM, we generated read counts for various combinations of parameters *e*, *p*, *n*, and *N* by assuming Hardy-Weinberg equilibrium. To mimic real situations, the setup of parameters we considered are as follows:

- *Mapping/sequencing error rate *e**. The mapping/sequencing error rate *e* across the genome was generated from a truncated normal distribution, i.e. *e* ∼ max{0, *N*(*μ*, *σ* ^2^)}, where *μ* was either 10^−2^ or 10^−3^, and *σ* ^2^ = 10^−6^.
- *MAF *p**. To mimic rare variants, less rare variants, and common variants, we generated *p* across the genome from the uniform distributions *U*(0.001, 0.01), *U*(0.01, 0.05), and *U*(0.05, 0.1), respectively. Futhermore, *p* = 0 for all non-SNPs.
- *Sample size *n**. We considered various sample sizes: 50, 100, 200, 500.
- *Read coverage *N**. The read coverage *N* across *n* samples follows the generalized Poisson distribution with expectation *μ* and the dispersion parameter λ defined by the expectation divided by the variance. This distribution reduces to the Poisson distribution if λ = 1, and a variance overdisperion is present if λ < 1. We considered various expectations: *μ* = 5, 10, 20, and 30, and fixed λ at 0.4. Here the dispersion parameter 0.4 was evaluated through two real NGS datasets from the “1000 genomes project” [8, 9].

Since the proportion of SNPs on the human genome is approximately 1%, we simulated read counts of 5 × 10^3^ SNP loci for each of 2 × 3 × 4 × 4 = 96 parameter combinations (*p* ≠ 0) and read counts of 5 × 10^5^ non-SNP loci for each of 2 × 1 × 4 × 4 = 32 parameter conbinations (*p* = 0).

### Sequence Data

We used NGS reads generated by the module *wgsim* in SAMtools to compare the performance of all considered SNP callers.

Given a reference sequence, *wgsim* can generate pair-end reads in fastq format. We extracted a reference sequence with a length of 1.9 × 10^6^ bp from the *chr20q13.32* region of human reference genome (version: GRCh37 of NCBI [25]). The parameters in *wgsim* were specified below:

- Base error rate = 0.001, 0.005, or 0.01.
- Number of read pairs = 6.8 × 10^4^, 1.4 × 10^5^, or 2.7 × 10^5^. These numbers corresponded to mean coverages 5, 10, and 20, respectively.

We adopted all default setting in *wgsim*: average read length = 70 bp, outer distance between two ends = 500, mutation rate = 0.001, percentage of SNPs among mutations = 85%. For each combination of base error rate and read number, we generated reads for 50 and 100 samples, separately, which resulted in 18 multi-sample datasets.

BWA [26] was used to align the simulated sequence reads onto the reference and to obtain a bam file for each sample. Multi-sample calling mode was used when calling SNPs using GATK and SAMtools. Read counts were extracted from the bam files, which were used as inputs of seqEM and MAFsnp. Algorithms implemented in MAQ was used to align simulated sequence reads and then to call SNPs. The detailed parameters for these softwares are given in S2 Method.

## Real Data

The 1000 Genomes Project (1KGP) [8] sequenced more than 1000 individuals of diverse populations, aiming at establishing by far the most detailed catalogue of human genetic variation. We utilized two groups of sequence data from 1KGP, i.e. the whole genome sequencing data of 156 randomly chosen Asian people (ftp://ftp.1000genomes.ebi.ac.uk/vol1/ftp/data/) and the targeted exon sequencing data of 110 Asian people (ftp://ftp.1000genomes.ebi.ac.uk/vol1/ftp/technical/pilot3_exon_targetted_GRCh37_bams/data/). All the samples were sequenced on Illumina platforms, the reads were aligned using BWA, and duplicates were removed using PICARD [21]. A brief description of the datasets is given to Table 1. We used a fragment of length 500Kb on chromosome 20 (position: 57,500,001 ∼ 58,000,000) for both whole genome data and target exon sequence data. This fragment also covers the ‘chr20q13.32’ region used in the simulated sequence data. A simple read filter was performed before seqEM and MAFsnp were applied to the data. To use MAQ, all downloaded files were firstly converted to fastq files using ‘bam2fastq’ (http://gsl.hudsonalpha.org/information/software/bam2fastq) and alignment was performed using MAQ. On the other hand, the multi-sample calling modes of GATK and SAMtools used bam files as input. For comparison, we utilized dbSNP build 137 as a standard reference.

**Table 1:** Description of real datasets (1000 Genomes Project).

	**Size**	**Population (sample size)**	**Mean coverage**
Whole genome	156	CHS(71),CDX(3),CHB(49),JPT(33)	4.7×
Targeted exon	110	CHD(32),CHB(17),JPT(61)	2.4×

CHB, Han Chinese; CDX, Dai Chinese; CHD, Denver Chinese; CHS, Southern Han Chinese; JPT, Japanese.

## Simulation results

### Read count data

**Fig 1:**
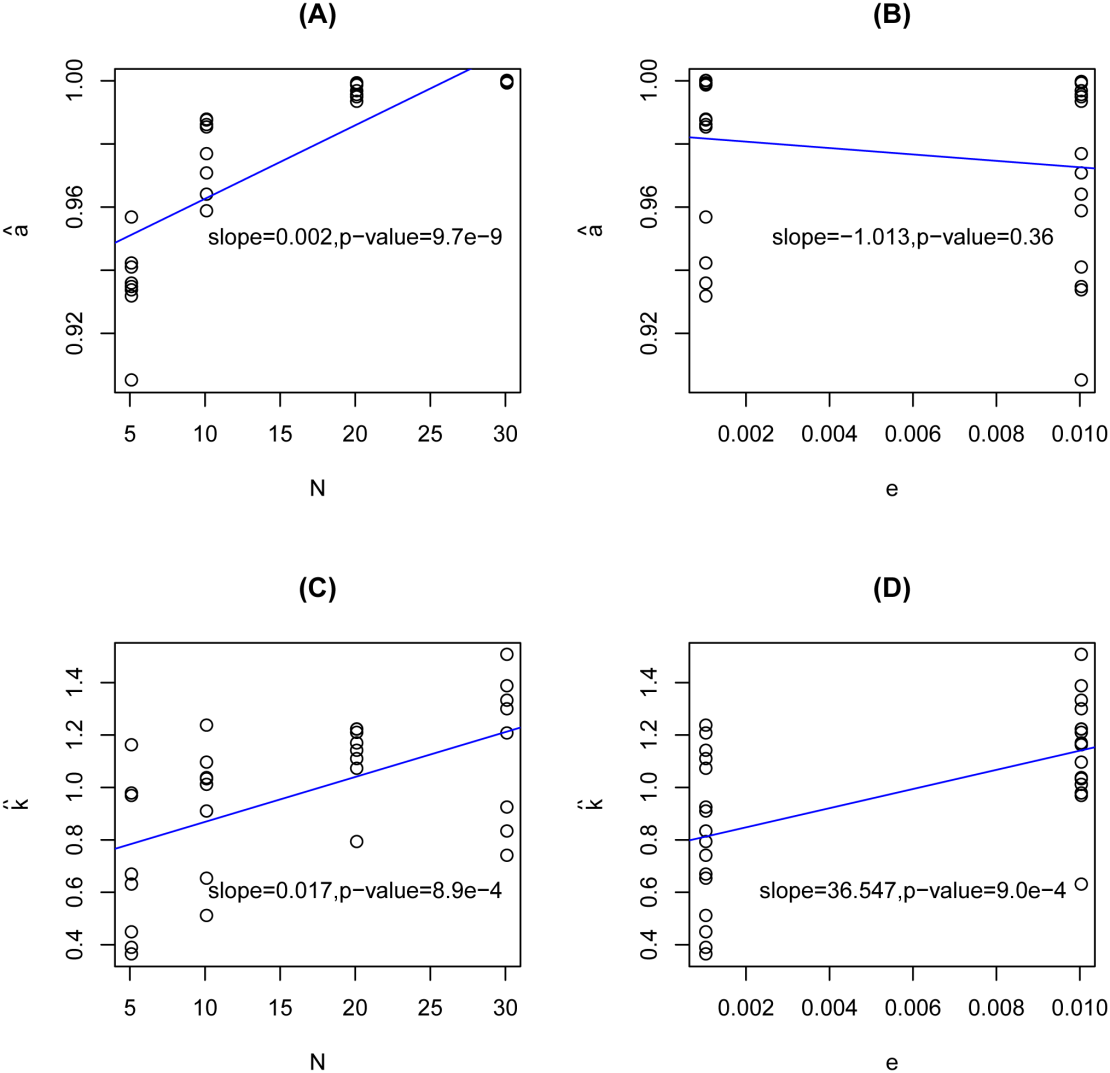
The relationship between ($\hat{a}$, $\hat{k}$) and (mean coverage *N*, error rate *e*). (A) Scatterplot of $\hat{a}$ vs. *N*; (B) Scatterplot of $\hat{a}$ vs. *e*; (C) Scatterplot of $\hat{k}$ vs. *N*; (D) Scatterplot of $\hat{k}$ vs. *e*.

First we evaluate the finite sample null distribution of *T*_*j*_, i.e. *D*_*a*,*k*_. Accurate estimates of *a* (zero proportion of *T*_*j*_) and *k* (expectation of non-zero part of *T*_*j*_) are essential in calculating the p-value for SNP calling by MAFsnp. The distribution of the non-zero part of *T*_*j*_ is compared with the chi-square distribution $\chi_{1}^{2}$ via a Q-Q plot (S1 and S2 Figs). It is seen that $\chi_{1}^{2}$ fits *T*_*j*_ pretty well when the mapping/sequencing error rate *e* = 0.01 across all considered coverages and sample sizes. With a small mapping/sequencing error rate (*e* = 0.001) and a low coverage (*N* = 5), the fitting gets poorer. This might be due to the fact that the counts are too small in such situation. Actually, the distribution of *T*_*j*_ becomes rather dicrete as shown in the Q-Q plot. The estimated *a* and *k*, i.e., $\hat{a}$ and $\hat{k}$, are presented in S1 Table. In all situations, the $\hat{a}$’s are much greater than the theoretical value 0.5, with a mean value of 0.977. This result coincides with Theorems 1 and 2 in S1 Method. It is also noted that $\hat{a}$ is increasing in the coverage *N*, as shown in Fig 1(A). This is easy to explain: as the coverage *N* increases, it is more likely that 0 is the maximizer of the pseudo likelihood function under the null hypothesis *H*_0_:*p* = 0, which results in a larger *a*. The correlation between $\hat{a}$ and *e* is not that evident (Fig 1(B)). On the other hand, $\hat{k}$ has a mean value of 0.976 across the 32 datasets, which is very close to the theoretical value 1. We observe an evident increasing trend of $\hat{k}$ in both coverage *N* and error rate *e*, see Fig 1(C) and 1(D) for details. In summary, the approximated distribution *D*_*a*,*k*_ of *T*_*j*_ greatly deviates from the limiting one *D*_0.5,1_.

**Fig 2:**
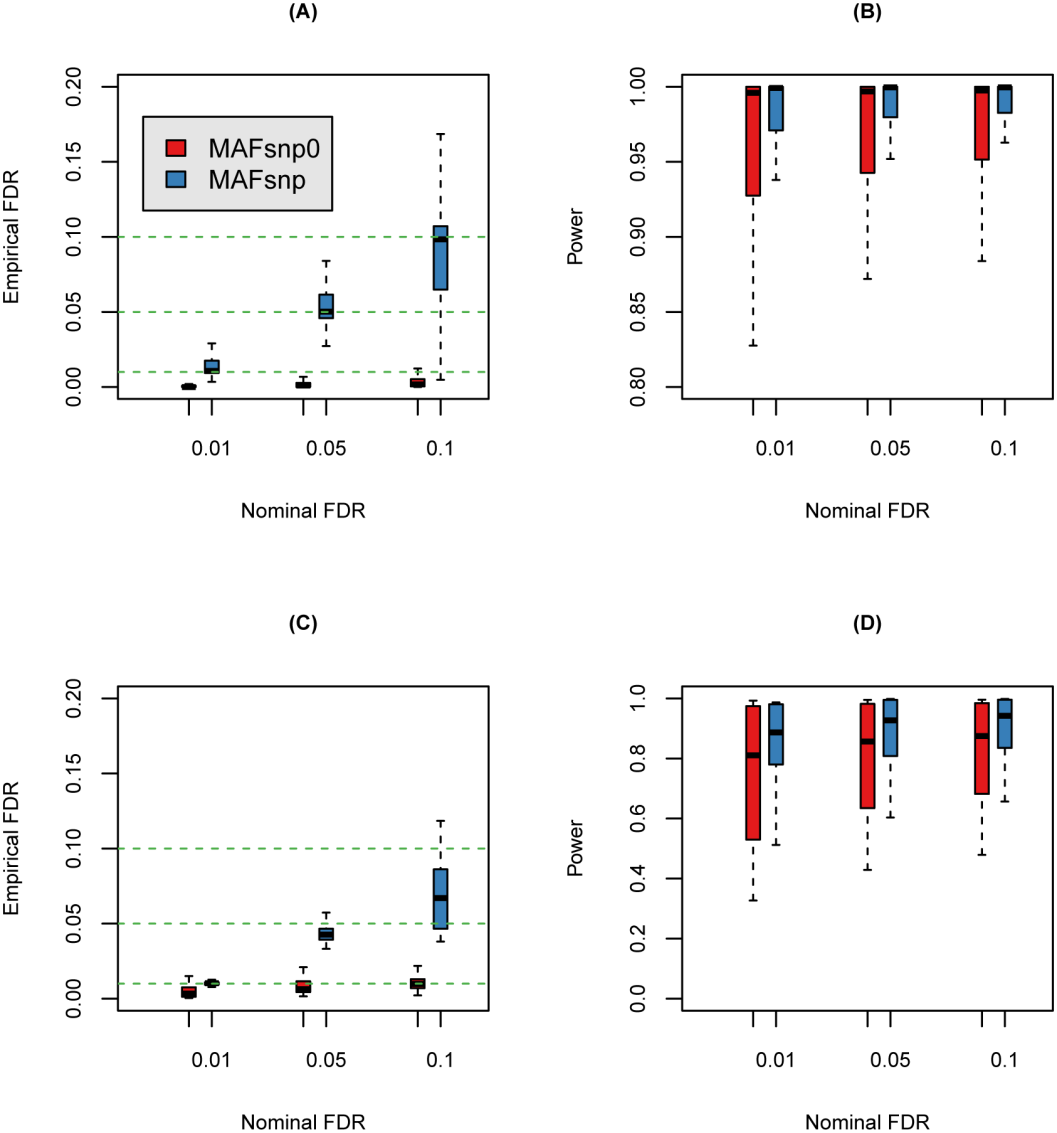
Boxplot of FDRs and powers for MAFsnp0 and MAFsnp at nominal FDR level *α* = 0.01, 0.05, 0.1. (A) Boxplot of FDRs with 96 read count datasets; (B) Boxplot of powers with 96 read count datasets; (C) Boxplot of FDRs with 18 sequence read datasets; (D) Boxplot of powers with 18 sequence read datasets.

Next we compare the performance of MAFsnp0 and MAFsnp in terms of FDRs and powers. The boxplot of FDRs and powers across 96 simulated datasets are presented in Fig 2(A) and 2(B). It is seen that the empirical FDRs of MAFsnp are virtually close to the nominal levels (Fig 2(A)), with median values being 0.011, 0.050, and 0.098 at nominal levels 0.01, 0.05, and 0.1, respectively. On the other hand, the empirical FDRs of MAFsnp0 are much smaller than the nominal levels, with median values being 0.000, 0.001, and 0.002 at nominal levels 0.01, 0.05, and 0.1, respectively. This indicates that MAFsnp0 is quite conservative. As a result, MAFsnp is much more powerful than MAFsnp0 (Fig 2(B)).

**Fig 3:**
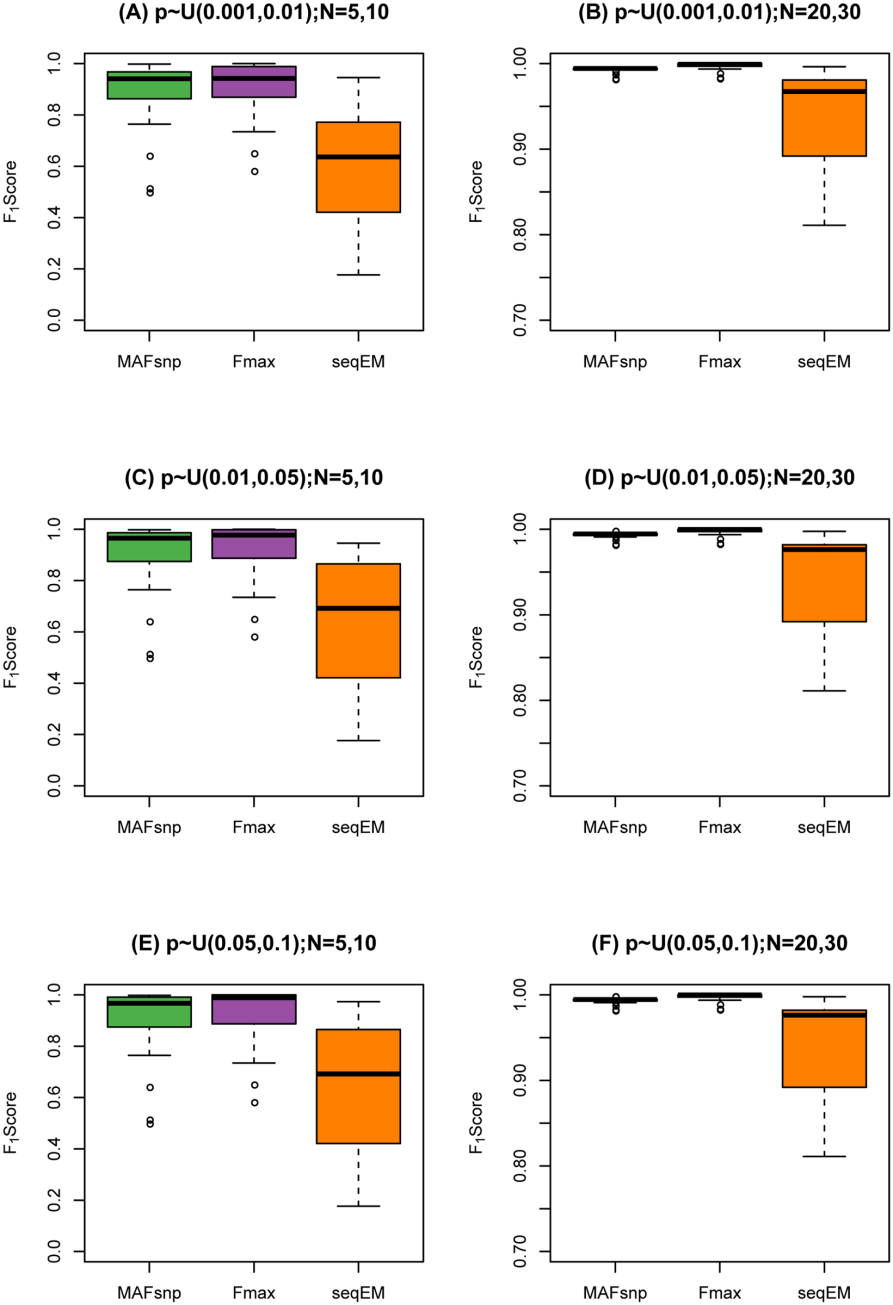
*F*_1_ scores of MAFsnp (*α* = 0.01) and seqEM for read count data.

Finally we compare the *F*_1_ scores of MAFsnp (nominal level = 0.01) and seqEM. For comparison purpose, the maximal *F*_1_ value on the precision-recall curve, i.e., *F*_*max*_, is calculated. All *F*_1_ values are displayed in Fig 3. It is seen that the *F*_1_ values of MAFsnp are only slightly smaller than *F*_*max*_, and MAFsnp almost uniformly outperforms seqEM.

### Sequence Data

Similar to the analysis of simulated read count data, we first check the estimation results for *D*_*a*,*k*_. S3–S5 Figs show how well the fitting of chi-square distribution is for various parameters combinations, and S2 Table gives the detailed estimates of *a* and *k*. It is seen that $\chi_{1}^{2}$ fits *T*_*j*_ very well with *e* = 0.005 or 0.01, while the fitting is slightly poor with *e* = 0.001. These results are in accordance with those for the read count data. As is seen in S2 Table, $\hat{a}$ has a mean value of 0.978, which is again much greater than the limiting value 0.5; on the other hand, $\hat{k}$ is generally greater than the theoretial value 1, especially when the coverage *N* gets larger.

Then we compare the empirical FDRs and powers of MAFsnp and MAFsnp0. As is shown in Fig 2(C) and 2(D), MAFsnp0 is quite conservative while MAFsnp has a much better control of FDR. For example, at nominal level 0.05, the median empirical FDR of MAFsnp0 is 0.006 while that of MAFsnp is 0.043. Again, MAFsnp is much more powerful than MAFsnp0. For example, at nominal level 0.05, the median power of MAFsnp is 0.927, which is much higher than 0.857 for MAFsnp0.

In what follows, we compare the results of the SNP callers SAMtools, GATK, MAQ, seqEM, and MAFsnp at nominal level 0.01. The empirical FDRs, powers, and F-scores are presented in S3–S5 Tables. MAFsnp has empirical FDRs well controlled around the nominal level 0.01, with a median value 0.010. The empirical FDRs of SAMtools and MAQ are much smaller, with median values 2.0 × 10^−5^ and 3.1 × 10^−3^, respectively. On the other hand, GATK and seqEM have much larger FDRs, with median values 0.182 and 0.155, respectively. Next we compare the powers of the considered SNP callers. SeqEM ranks the first with a median power of 0.955, but it has much larger FDRs as a trade off. MAFsnp ranks the second, with a median power value of 0.885. MAQ and SAMtools comes the next due to smaller FDRs, with median powers 0.855 and 0.775, respectively. Surprisingly, GATK is least powerful with a median power of 0.775, though it has the largest FDRs in general. Finaly, we compare the *F*_1_ scores that balance precisions and recalls. MAFsnp has the highest median *F*_1_ score of 0.935, which is close to *F*_*max*_ (= 0.945). SeqEM, SAMtools and GATK have lower *F*_1_ scores (median values are 0.920, 0.890, 0.875, and 0.785, respectively) due to either too small FDRs or too large FDRs. In summary, MAFsnp has empirical FDRs well controlled around the nominal level. Meanwhile, MAFsnp has the best balance of precisions and recalls.

## Real Data Applications

For real data, we do not know which variants are true SNPs, so the comparison metrics would be different from those for simulation data. Following [21], we considered three comparison metrics: 1) number of called SNPs, 2) proportion of called SNPs in dbSNP (build 137), and 3) Ti/Tv ratio for called SNPs. The second metric is referred to as *calling accuracy* from now. We fixed the nominal level at 0.01 in MAFsnp.

### Whole Genome Sequencing Data of 156 Asian People

This dataset has an overall mean coverage of about 4.7×, which is a classic representation of low coverage data. The SNP calling results of seqEM, MAQ, GATK, SAMtools, and MAFsnp are reported in Table 2. GATK, SAMtools and MAFsnp called comparable numbers of SNPs (around 1700 SNPs). MAFsnp has a higher calling accuracy (91.2%) than GATK (90.3%) and SAMtools (88.8%). Particularly, of 1699 SNPs called by MAFsnp, 1550 were found in dbSNP, while GATK called 10 more SNPs than MAFsnp but only 1544 were found in dbSNP. As previously mentioned, Ti/Tv ratio is a useful index in real sequence data analysis, and a higher Ti/Tv ratio is an indicator of a lower false positive rate. SAMtools has the largest Ti/Tv ratio, which is 2.329 for all called SNPs, 2.453 and 1.492 for known and novel SNPs, respectively. MAFsnp ranks the next, with a Ti/Tv ratio of 2.299 for all called SNPs, and 2.422 and 1.403 for known and novel SNPs, respectively. GATK has a smaller Ti/Tv ratio than SAMtools and MAFsnp. Compared with GATK, SAMtools, and MAFsnp, the other two SNP callers seqEM and MAQ called more SNPs (5351 and 2768, respectively) but had much lower calling accuracies (31.7% and 59.0%, respectively). Furthermore, seqEM and MAQ have much smaller Ti/Tv ratios than GATK, SAMtools, and MAFsnp. In summary, MAFsnp achieves the best balance between calling accuracy.

**Table 2:** SNP calling results of seqEM, MAQ, GATK, SAMtools, and MAFsnp for the 1000 Genomes Project data.

		Number of called SNPs			CA(%)^a^	Ti/Tv Ratio^b^		
		All	Known	Novel		All	Known	Novel
*Whole genome*	seqEM	5351	1698	3653	31.7	1.232	2.225	0.878
	MAQ	2768	1632	1136	59.0	1.582	2.372	0.889
	GATK	1709	1544	165	90.3	2.276	2.393	1.429
	SAMtools	1762	1564	198	88.8	2.329	2.453	1.592
	MAFsnp^c^	1699	1550	149	91.2	2.299	2.422	1.403
*Targeted exon*	seqEM	950	348	602	36.6	1.263	2.48	0.612
	MAQ	654	356	298	54.4	1.389	2.594	0.383
	GATK	171	156	15	91.2	1.803	2.12	0.364
	SAMtools	585	433	152	74.0	1.763	2.305	0.875
	MAFsnp^c^	470	405	65	86.2	1.749	2.375	0.275

^*a*^Calling accuracy;

^*b*^transition/transversion ratio;

^*c*^nominal FDR level = 0.01.

### Targeted Exon Sequencing Data of 110 Asian People

The average coverage is 2.4× for this targeted exon sequencing dataset. The SNP calling results are again presented in Table 2. As in the whole genome dataset, seqEM and MAQ called more SNPs than the other three methods, but had much lower calling accuracy and Ti/Tv ratio. GATK called least SNPs, with a number of only 171, compared with 585 by SAMtools and 470 by MAFsnp. Although both calling accuracy and Ti/Tv ratio are highest, GATK is evidently most conservative. MAFsnp called 470 SNPs, which is smaller than 585 by SAMtools, but the calling accuracy of MAFsnp (86.2%) is much higher than that of SAMtools (74.0%). Although the overall Ti/Tv ratio of MAFsnp, 1.749, is slighly lower than that of SAMtools, the Ti/Tv ratio of MAFsnp for known SNPs is slightly higher. Overall, in this dataset, MAFsnp again achieves the best balance between number of called SNPs, calling accuracy, and Ti/Tv ratio.

## Discussion

Sequencing a large sample of individuals is a trend in NGS studies. Most existing SNP callers are based on Bayes frameworks, which cannot control FDR at desired nominal levels. We propose a novel multiple-sample SNP caller “MAFsnp” based on a likelihood model. In MAFsnp, the SNP calling issue is transformed into a hypothesis testing problem in a frequentist framework, so that a list of p-values can be obtained, which can be used to call SNPs by controlling FDR at any given nominal level.

In this article, we propose a new SNP caller MAFsnp by using a eLRT statistic and approximating the null distribution of the statistic with a novel distribution *D*_*a*,*k*_. The simulation results of both read count data and sequence data demonstrate that the new distribution *D*_*a*,*k*_ has a much better control of FDRs compared with the conventional limiting distribution *D*_0.5,1_. The performance of MAFsnp is compared with those of some existing SNP callers through both simulated data and two real datasets. For the simulated read count data, MAFsnp outperforms seqEM in almost all situations; for the simulated sequence data, MAFsnp has a better balance of precisions and recalls than SAMtools, GATK, seqEM, and MAQ. In the application to two real datasets with low coverage, MAFsnp is demonstrated to have a better performance compared with the other SNP callers and achieves a good balance between the number of called SNPs, calling accuracy, and Ti/Tv ratio.

MAFsnp has several features. First, MAFsnp is the first NGS data based SNP caller that provides p-values for calling SNPs. Second, a pseudo-likelihood function is adopted to greatly speed up calling speed. Third, a novel distribution *D*_*a*,*k*_ is proposed to approximate the null distribution of the eLRT statistic. Forth, MAFsnp is based on read count data, making it applicable to all types of sequence data. Fifth, MAFsnp avoids a tedious filtering procedure used in Bayesian methods.

The proposed distribution *D*_*a*,*k*_ has potentially wide application areas in many biologic research circumstances where associated parameters are on/near the boundary of parameter space. For example, in genetic association studies, the question of interest is to compare the allele frequencies between cases and controls. For rare variants, the allele frequencies are close to zero, i.e, the boundary of the parameter space. The distribution *D*_*a*,*k*_ could searve as a good null distribution of the conventional test statistics for association testing. The Hardy-Weinberg equilibrium is assumed in MAFsnp, which uses a single parameter (i.e. MAF *p*) to characterize the genotype frequencies. This assumption could be relaxed by introducing two parameters, i.e., the frequencies of *RR* and *Rr*, denoted by *p*_*RR*_ and *p*_*Rr*_. The corresponding hypothesis testing problem can be modified as *H*_0_ : *p*_*RR*_ = 1 versus *H*_1_ : *p*_*RR*_ < 1.

The speed of MAFsnp depends on coverage, sample size, and number of nucleotide loci. For the whole genome dataset analyzed in this article (average coverage was ∼ 4.7×, sample size was 156), it took a 3.20GHz CPU laptop computer about 2.5 minutes to call SNPs from 500k nucleotide loci. It could take a longer time to call SNPs when the covarage gets lower. For the targeted exon dataset we analyzed (average coverage was ∼ 2.4×, sample size was 110), it took the computer 18 minutes to call SNPs from 500k nucleotide loci. An R package implementing MAFsnp is available publicly at http://homepage.fudan.edu.cn/zhangh/softwares/.

## Supporting Information

S1 Method Theoretical property for the null distribution of eLRT statistic. (PDF)

S2 Method Parameter setting of existing SNP callers. (PDF)

S1 FigQ-Q plot of non-zero *T*_*j*_ under null hypothesis vs. the $\chi_{df=1}^{2}$ distribution (simulated read count data, *e* = 0.001).Red straight line has a slope $\hat{k}$.(PDF)

S2 FigQ-Q plot of non-zero *T*_*j*_ under null hypothesis vs. the $\chi_{df=1}^{2}$ distribution (simulated read count data, *e* = 0.01).Red straight line has a slope $\hat{k}$.(PDF)

S3 FigQ-Q plot of non-zero *T*_*j*_ under null hypothesis vs. the $\chi_{df=1}^{2}$ distribution (simulated sequence data, *e* = 0.001).Red straight line has a slope $\hat{k}$.(PDF)

S4 FigQ-Q plot of non-zero *T*_*j*_ under null hypothesis vs. the $\chi_{df=1}^{2}$ distribution (simulated sequence data, *e* = 0.005).Red straight line has a slope $\hat{k}$.(PDF)

S5 FigQ-Q plot of non-zero *T*_*j*_ under null hypothesis vs. the $\chi_{df=1}^{2}$ distribution (simulated sequence data, *e* = 0.01).Red straight line has a slope $\hat{k}$.(PDF)

S1 Table Estimates of *a* and *k* by MAFsnp (simulated read count data). (PDF)

S2 Table Estimates of *a* and *k* by MAFsnp (simulated sequence data). (PDF)

S3 Table False discovery rates of considered SNP callers (simulated sequence data). (PDF)

S4 Table Powers of considered SNP callers (simulated sequence data). (PDF)

S5 Table F-scores of considered SNP callers (simulated sequence data). (PDF)
