# Supplementary material for: MAFsnp: A Multi-Sample Accurate and Flexible SNP Caller Using Next-Generation Sequencing Data
Source: PLoS One. 2015 Aug 26;10(8):e0135332. doi: 10.1371/journal.pone.0135332 (PMC4550471; doi:10.1371/journal.pone.0135332)
Supplement: S1 Method — (PDF) [file pone.0135332.s001.pdf]

Theoretical property of the null distribution of LRT

Here we show, under the null distribution, that the proportion of zero part in the LRT statistic could greatly deviate from the asymptotic result $D_{0.5,1}$, see Theorem 1 (constant N) and Theorem 2 (non-constant N) for details.

Constant N

Recall that the log-likelihood for the jth locus is $l(e,p) = l_j\big((e,p), \mathbf{X}_j, \mathbf{N}_j\big)$. To simplify notation, the indices j are omitted in e_j, p_j, $\mathbf{X}_j$, $X_{i,j}$, $\mathbf{N}_j$, and $N_{i,j}$. Suppose $(e_0, 0)$ is the true value of (e,p), then the derivative of $l(e,p)$ evaluated at $(e_0, 0)$ is

$$\frac{\partial l(e_0,0)}{\partial p} = 2\sum_{i=1}^{n}\left\{2^{-N_i} e_0^{-X_i}(1-e_0)^{X_i-N_i} - 1\right\}, \tag{e1}$$

whose expectation is equal to

$$\begin{aligned}
\mathrm{E}\left\{\frac{\partial l(e_0,0)}{\partial p}\right\} &= 2n\sum_{m=1}^{\infty}\sum_{j=0}^{m}\left\{\left[\frac{(\frac{1}{2})^m}{e_0^j(1-e_0)^{m-j}} - 1\right]\Pr(X=j|N=m)\Pr(N=m)\right\} \\
&= 2n\sum_{m=1}^{\infty}\Pr(N=m)\sum_{j=0}^{m}\binom{m}{j}[2^{-m} - e_0^j(1-e_0)^{m-j}] = 0
\end{aligned}$$

Denote by $(\hat{e},\hat{p})$ the maximizer of $l(e,p)$. It is easy to see that $\tilde{e} = \sum_{i=1}^n X_i/\sum_{i=1}^n N_i$ is the maximizer of $l(e,0)$. We have the following lemma:

Lemma 1.

$$\begin{aligned}
&\Pr[\partial l(\tilde{e},0)/\partial p < 0] \\
\leq &\Pr\left[(\tilde{e},0) \textit{ is a strict local maximizer of } l(e,p)\right] \\
\leq &\Pr[\partial l(\tilde{e},0)/\partial p \leq 0].
\end{aligned}$$

Proof. If $(\tilde{e},0)$ is a strict local maximizer of $l(e,p)$, there there exists $\delta > 0$, such that for any $\epsilon \in [0,\delta)$, $l(\tilde{e},0) - l(\tilde{e},\epsilon) \geq 0$. Therefore,

$$\frac{\partial l(\tilde{e},0)}{\partial p} = \lim_{\epsilon \downarrow 0}\frac{l(\tilde{e},\epsilon) - l(\tilde{e},0)}{\epsilon} \leq 0.$$

On the other side, given $\partial l(\tilde{e},0)/\partial p < 0$,

$$\begin{aligned} l(e,p) =& l(\tilde{e},0) + [\partial l(\tilde{e},0)/\partial e](e-\tilde{e}) + [\partial l(\tilde{e},0)/\partial p](p-0)[1+o(|p|+|e-\tilde{e}|)] \\ &+ \frac{\partial^2 l(\tilde{e},0)}{\partial e^2}(e-\tilde{e})^2[1+o(|e-\tilde{e}|)] \end{aligned}$$

for (e,p) in a neighborhood of $(\tilde{e},0)$. By $\partial l(\tilde{e},0)/\partial e = 0$, $\partial l(\tilde{e},0)/\partial p < 0$, and

$$\frac{\partial^2 l(\tilde{e},0)}{\partial e^2} = \sum_{i=1}^{n} -X_i/e^2 - (N_i - X_i)/(1-e)^2 < 0,$$

we have $l(e,p) < l(\tilde{e},0)$ for (e,p) in some neighborhood of $(\tilde{e},0)$. Lemma 1 is proved.

Lemma 2.

$$\Pr(\frac{\partial l(\tilde{e},0)}{\partial p} < 0) \leq \Pr(T=0) \leq \Pr(\frac{\partial l(\tilde{e},0)}{\partial p} \leq 0),$$

where T is the likelihood ratio test statistic defined in (3) or (4) of the main text.

Note that $\Pr(T=0) = \Pr(\hat{p}=0)$, Lemma 2 follows immediately from Lemma 1.

It should be noted that the above lemmas are suitable for both small sample size and large sample size. As stated in Self and Liang (1987), T should be asymptotically distributed as $D_{0.5,1}$, which implies

$$\Pr(T=0) \to 0.5. \tag{e2}$$

In what follows, we show that Lemma 2 also implies (e2). From Lemma 2, by virtue of Linderberg's Central Limit Theorem under H_0, as $n \to \infty$, we have that both $n^{-1/2}\partial l(e_0,0)/\partial p$ and $\sqrt{n}(\tilde{e}-e_0)$ converge to normal distributions so that

$$\Pr\left(\frac{\partial l(\tilde{e},0)}{\partial p} < 0\right) \to 0.5 \text{ and } \Pr\left(\frac{\partial l(\tilde{e},0)}{\partial p} \leq 0\right) \to 0.5,$$

which implies (e2) by Lemma 2.

Now we examine the small sample property of $\hat{p}$ with constant N. Before stating Theorem 1, we assume the following conditions hold:

$$K \in \{0, 1, \cdots, m\},\ a_j \in \mathbb{Z}_+,\ \sum_{j=0}^{K} a_j = n,\ \sum_{j=0}^{K} a_j g(\frac{\sum_{k=0}^{K} k a_k}{mn}, m, j) < 0, \tag{C-1}$$

$$K \in \{0, 1, \cdots, m\},\ a_j \in \mathbb{Z}_+,\ \sum_{j=0}^{K} a_j = n,\ \sum_{j=0}^{K} a_j g(\frac{\sum_{k=0}^{K} k a_k}{mn}, m, j) \leq 0. \tag{C-2}$$

Define the function

$$g(e, m, j) = \frac{0.5^m}{e^j (1-e)^{m-j}} - 1 \text{ for } g \in [0, 0.5] \times \mathbb{N}_+^2$$

and the sets

$$A_k(n, m) = \{(K, \boldsymbol{a} = (a_0, a_1, \cdots, a_K)^\tau) | K, a_j \text{ satisfies condition (C-}k\text{) for } j = 0, 1, \cdots, K\}, k = 1, 2.$$

Applying Lemma 2, we have the following Theorem.

Theorem 1. *Given a fixed integer m, under H_0, we have* $\Pr(T = 0 | N_i = m) \in$

$$\left[\sum_{(K,\boldsymbol{a}) \in A_1(n,m)} \binom{n}{a_0\ a_1\ a_2\ \cdots\ a_K} \prod_{j=0}^{K} B(j, m, e_0)^{a_j}, \sum_{(K,\boldsymbol{a}) \in A_2(n,m)} \binom{n}{a_0\ a_1\ a_2\ \cdots\ a_K} \prod_{j=0}^{K} B(j, m, e_0)^{a_j} \right],$$

where $B(j, m, e_0)$ is defined in (2) of the main text.

Based on Theorem 1, we calculated the upper and lower bounds of $\Pr(T = 0 | N_i = m)$ for $n = 20, 50, 100$, $e = 0.001, 0.01, 0.05$ and $m = 2, 5, 10, 20$. We found that the difference between the upper bound and lower bound was uniformally smaller than 10^{-5}. Therefore, we display in Figure 1 the mean value of the upper bound and lower bound (denoted by 'prob') for each parameter combination, which is decreasing in both n and e. These probabilities greatly deviate from the limiting value 0.05, especially when $e \leq 0.01$.

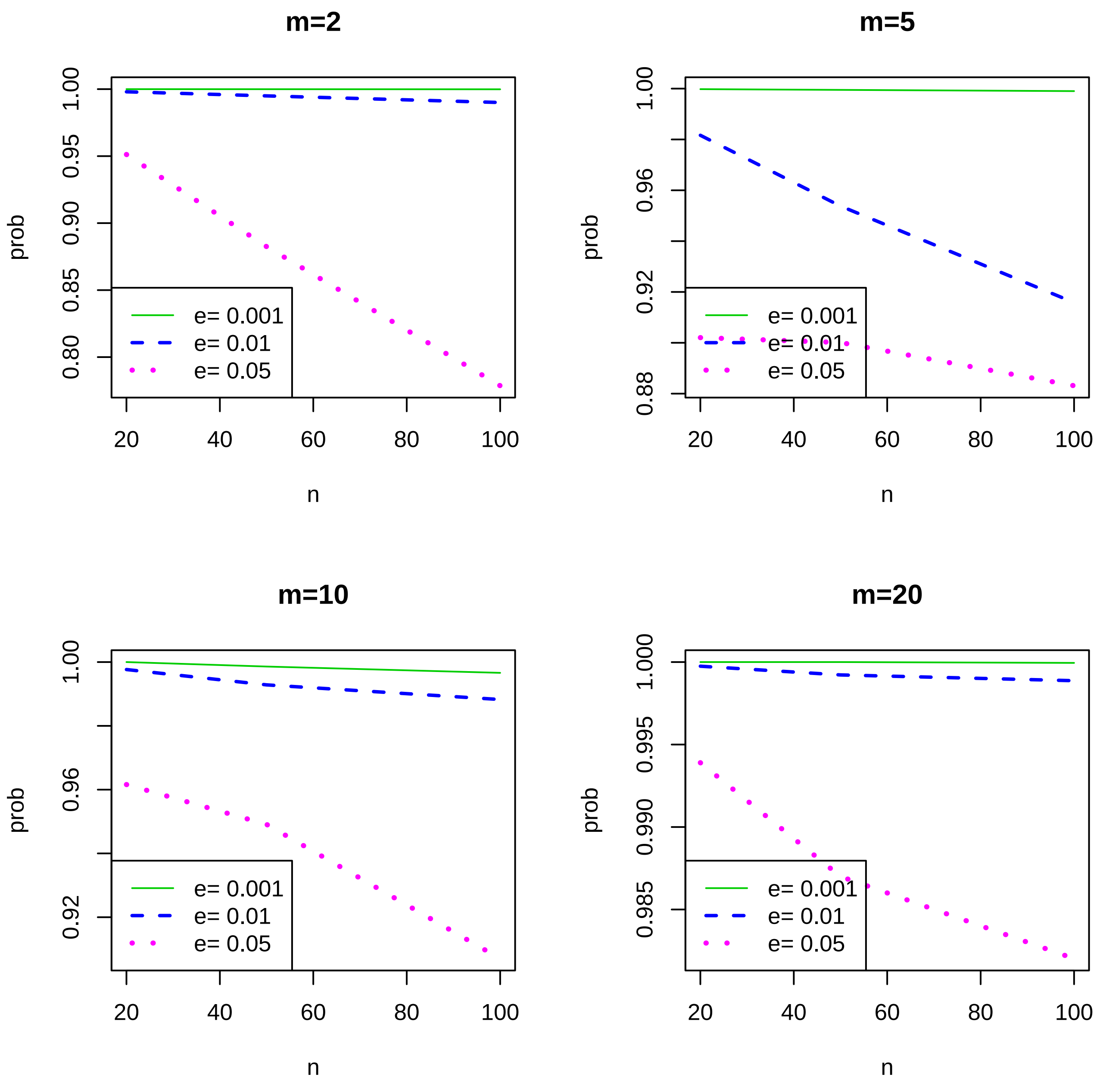

Figure 1: $\Pr(T = 0|N_i = m)$ under H_0 with constant N $(= m)$.

Non-constant N

Now we consider a non-constant N. We need the following condition.

$$v_j \in \mathbb{Z}_+,\ v_j \leq m_j,\ \text{for } j = 1, 2, \cdots, n;\ \sum_{j=0}^{n} g(\frac{\sum_{k=0}^{n} v_k}{\sum_{k=0}^{n} m_k}, m_j, v_j) < 0, \tag{C-3}$$

$$v_j \in \mathbb{Z}_+,\ v_j \leq m_j,\ \text{for } j = 1, 2, \cdots, n;\ \sum_{j=0}^{n} g(\frac{\sum_{k=0}^{n} v_k}{\sum_{k=0}^{n} m_k}, m_j, v_j) \leq 0 \tag{C-4}$$

Define the sets

$$U_k(n, \boldsymbol{m}) = \{\boldsymbol{v} = (v_1, v_2, \cdots, v_n)^\tau)|v_j \text{ satisfies condition (C-}k\text{) for } j = 0, 1, \cdots, n\}, k = 1, 2,$$

where $\boldsymbol{m} = (m_1, \cdots, m_n)^\tau$.

Similarly to Theorem 1, we have

Theorem 2. *If $N_1, N_2, \cdots, N_n$ are independently distributed as $\mathrm{GP}(\mu, \lambda)$, then the MLE $\hat{p}$ satisfies*

$$\Pr(T = 0) = \Pr(\hat{p} = 0) \in [\Pr(\partial l(\tilde{e}, 0)/\partial p < 0), \Pr(\partial l(\tilde{e}, 0)/\partial p \leq 0)],$$

where

$$\Pr(\partial l(\tilde{e}, 0)/\partial p < 0) = \prod_{k=1}^{m} \left[\frac{1}{m_k!} u^{m_k} (1 + a m_k)^{m_k - 1} e^{-u(1 + a m_k)} \sum_{\boldsymbol{v} \in U_1(n, \boldsymbol{m})} \prod_{j=0}^{n} B(v_j, m_j, e_0) \right],$$

$$\Pr(\partial l(\tilde{e}, 0)/\partial p \leq 0) = \prod_{k=1}^{m} \left[\frac{1}{m_k!} u^{m_k} (1 + a m_k)^{m_k - 1} e^{-u(1 + a m_k)} \sum_{\boldsymbol{v} \in U_2(n, \boldsymbol{m})} \prod_{j=0}^{n} B(v_j, m_j, e_0) \right],$$

and

$$u = \mu\sqrt{\lambda}, a = \frac{1}{u} - \frac{1}{\mu}.$$

Here $\mathrm{GP}(\mu, \lambda)$ is the generalized Poisson distribution with mean μ and variance μ/λ, whose probability function is

$$\Pr(N = k) = \frac{1}{k!} u^k (1 + ak)^{k-1} e^{-u(1+ak)}, u = \mu\sqrt{\lambda}, a = 1/u - 1/\mu.$$

Based on Theorem 2, we also calculated the upper and lower bounds of $\Pr(T = 0|N_i = m)$ for $n = 20, 50, 100$, $e = 0.001, 0.01, 0.05$ and $m = 2, 5, 10, 20$. We found a trend very similar to that for constant N.

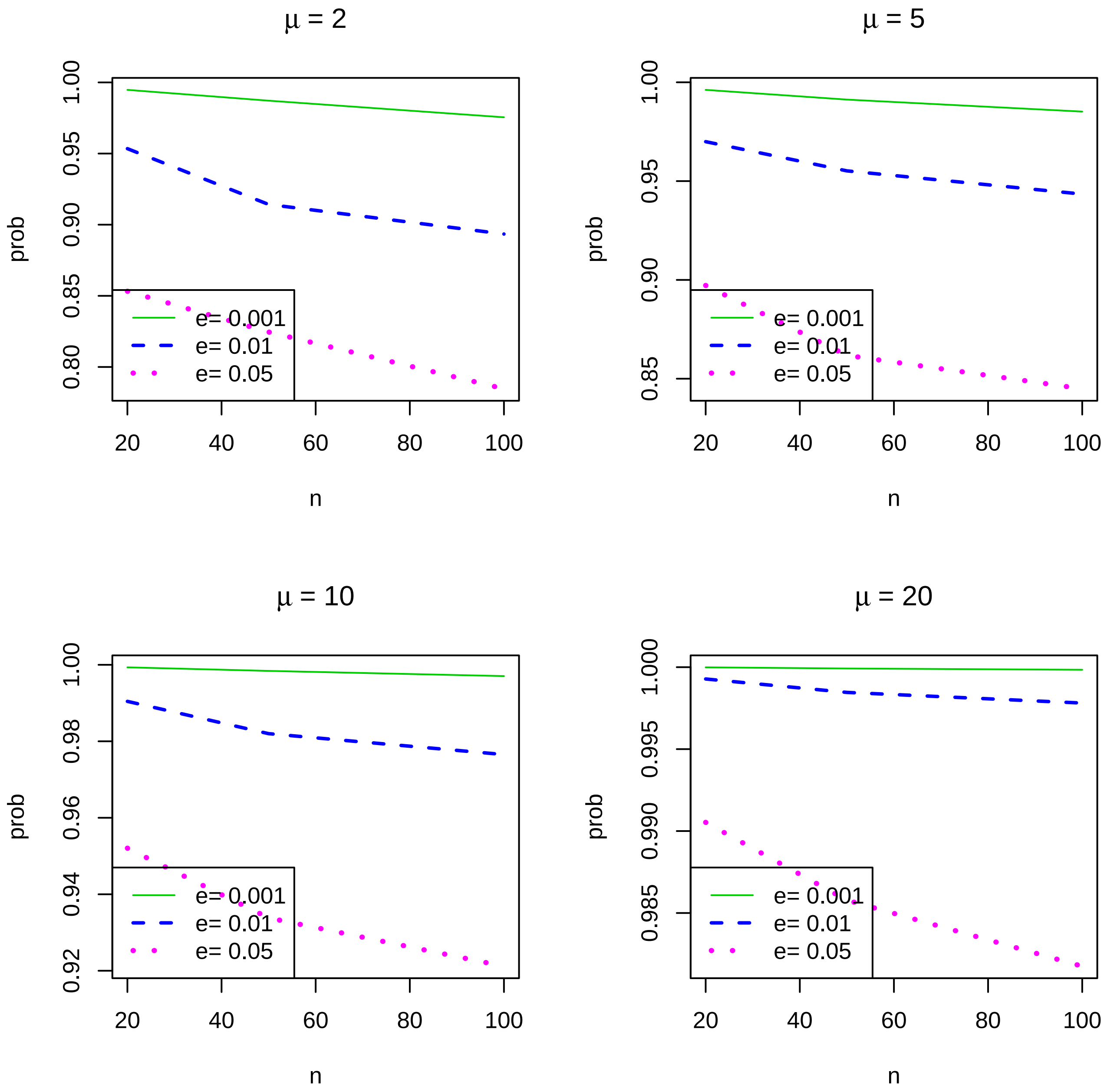

Figure 2: $\Pr(T=0)$ under H_0 when $N \sim \mathrm{GP}(\mu, 1)$.
