# Supplementary material for: MAFsnp: A Multi-Sample Accurate and Flexible SNP Caller Using Next-Generation Sequencing Data
Source: PLoS One. 2015 Aug 26;10(8):e0135332. doi: 10.1371/journal.pone.0135332 (PMC4550471; doi:10.1371/journal.pone.0135332)
Supplement: S2 Method — (PDF) [file pone.0135332.s002.pdf]

Parameter setting of SNP callers

- ***MAQ***. We conducted read alignment and SNP calling based on the MAQ workflow given in http://maq.sourceforge.net/maq-man.shtml. To filter reads, we used script ``maq.pl SNPfilter'' with parameters:

  1) -d 3: filter SNPs with coverage <3 for whole genome sequence and targeted exon data as the mean coverage of the two data sets are both low;

  2) - q 10 for N <10 and 20 for N >10. This is the consensus quality (Q_S) threshold.

- ***GATK***. GATK-HaplotypeCaller is highly recommended by the authors of GATK. HaplotypeCaller needs to do local de novo assembly, which is quite slow. We conducted SNP calling steps following the latest recommendations from the authors of GATK (https://www.broadinstitute.org/gatk/guide/best-practices?bpm=DNAseq#methods_vqsr51), with filtering commands: VariantFiltration --filterExpression “QD < 2.0 || FS > 60.0 || MQ < 40.0|| MappingQualityRankSum < -12.5|| ReadPosRankSum <-8.0”. Please check the website for details.

- ***SAMtools***. We used the ‘mcompile’ command for multi-sample SNP calling. Vcftools was used to filter SNPs, with filtering parameter “-D15000 and -d50”, which is the upper and lower bound for the total coverage of all samples.

- ***seqEM***. Default parameter was used to call genotypes and SNPs, except for the ‘min_read_depth’ parameter. We set it to be 3 in accord with MAQ. When transforming bam files into count files, we discarded those bases with base quality less than 53 and those reads with mapping quality less than 10. Variations around indels (±5) were also filtered out.
