# Supplementary figures and images for: MAFsnp: A Multi-Sample Accurate and Flexible SNP Caller Using Next-Generation Sequencing Data
Source: PLoS One. 2015 Aug 26;10(8):e0135332. doi: 10.1371/journal.pone.0135332 (PMC4550471; doi:10.1371/journal.pone.0135332)

N=5,n=50

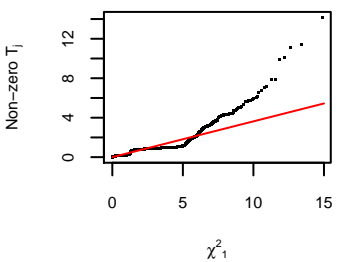

N=5,n=100

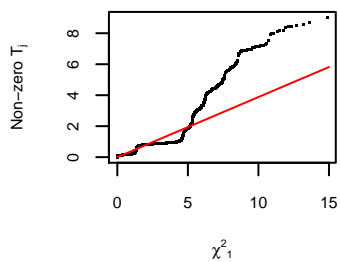

N=5,n=200

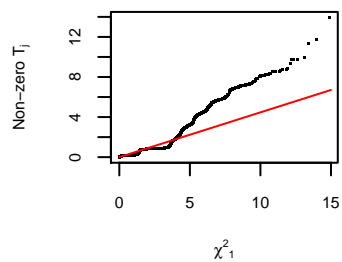

N=5,n=500

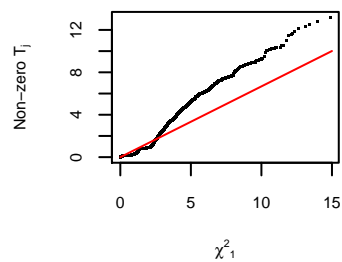

N=10,n=50

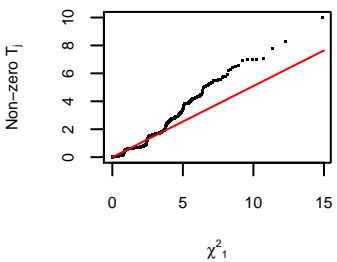

N=10,n=100

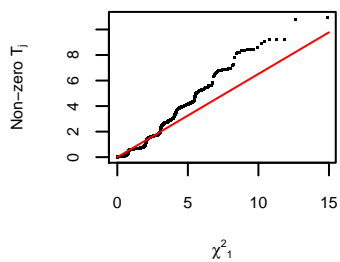

N=10,n=200

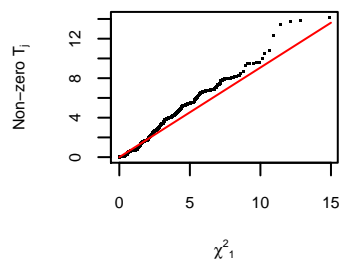

N=10,n=500

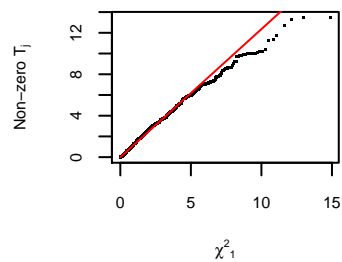

N=20,n=50

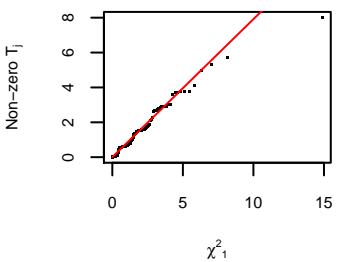

N=20,n=100

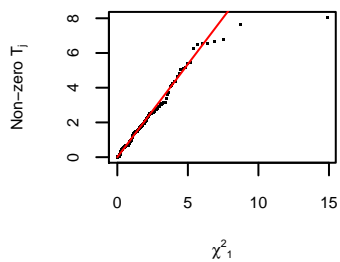

N=20,n=200

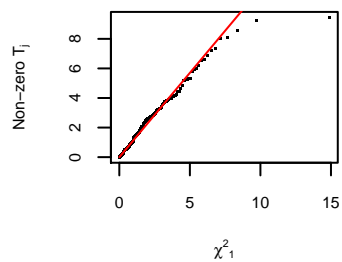

N=20,n=500

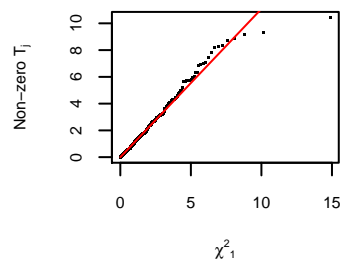

N=30,n=50

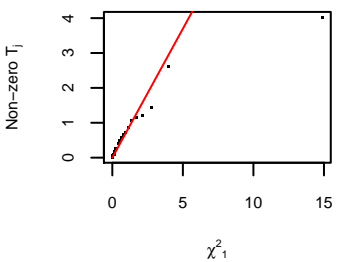

N=30,n=100

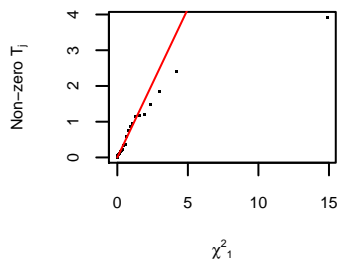

N=30,n=200

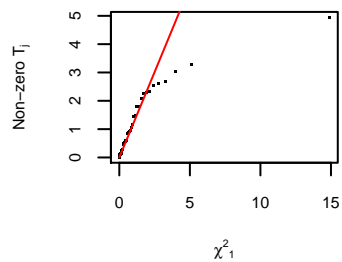

N=30,n=500

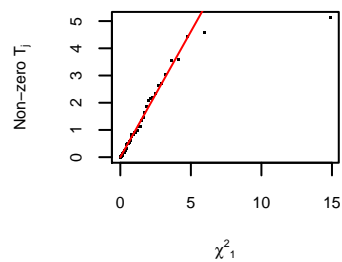

Supplement: S1 Fig — Red straight line has a slope k^. (PDF) [file pone.0135332.s003.pdf]

N=5,n=50

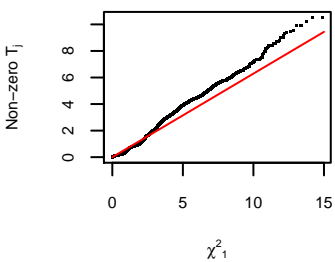

N=5,n=100

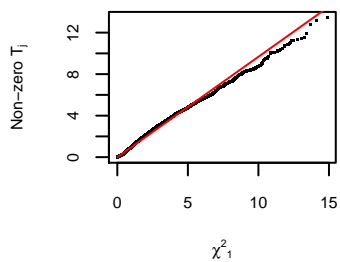

N=5,n=200

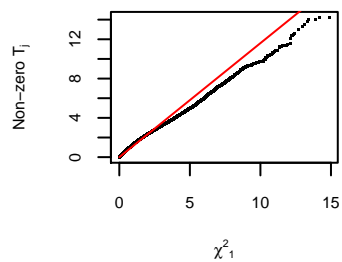

N=5,n=500

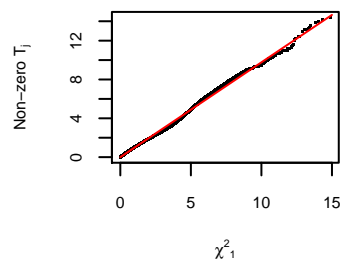

N=10,n=50

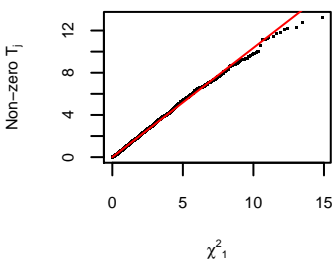

N=10,n=100

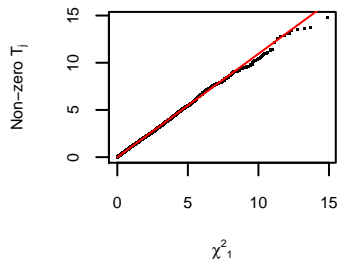

N=10,n=200

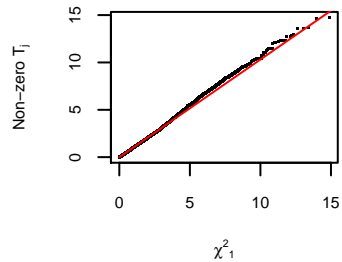

N=10,n=500

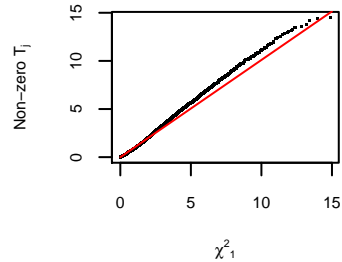

N=20,n=50

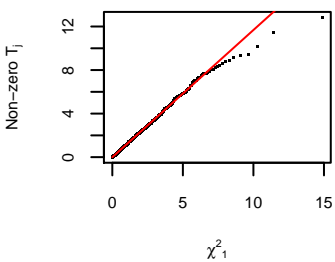

N=20,n=100

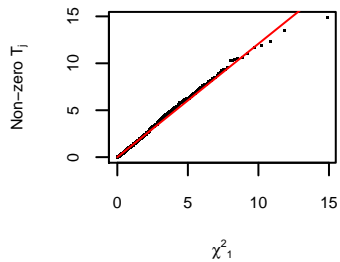

N=20,n=200

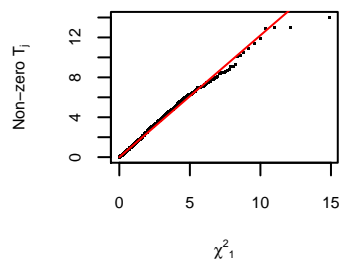

N=20,n=500

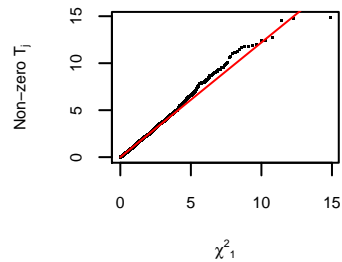

N=30,n=50

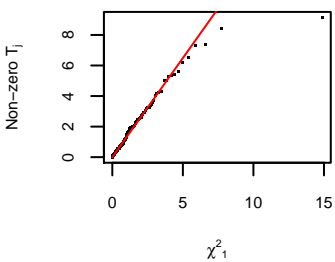

N=30,n=100

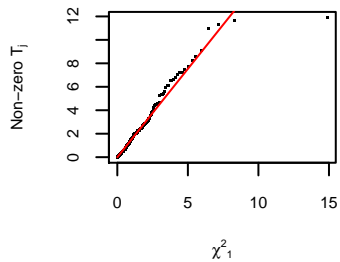

N=30,n=200

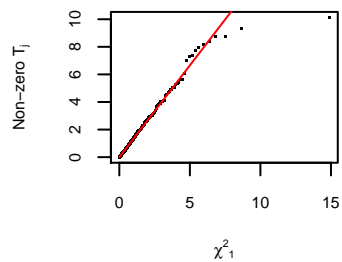

N=30,n=500

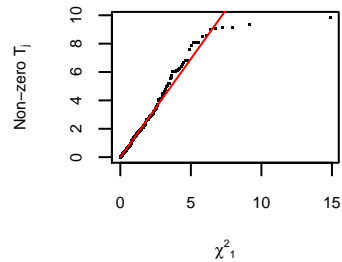

Supplement: S2 Fig — Red straight line has a slope k^. (PDF) [file pone.0135332.s004.pdf]

**N=5,n=50**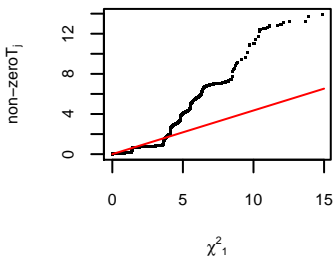**N=5,n=100**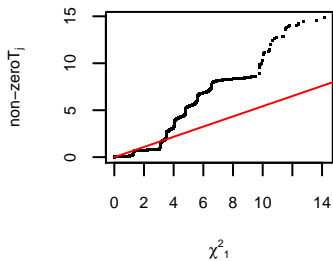**N=10,n=50**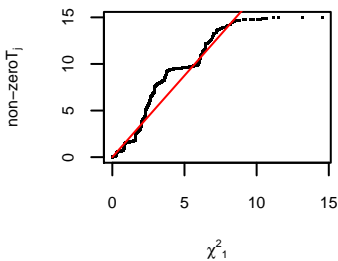**N=10,n=100**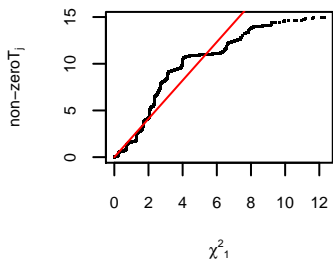**N=20,n=50**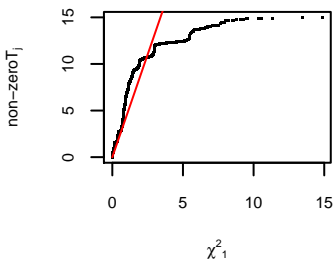**N=20,n=100**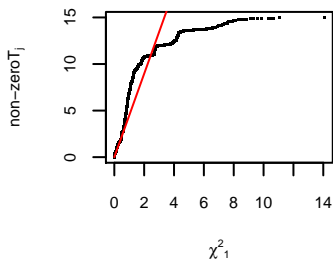

Supplement: S3 Fig — Red straight line has a slope k^. (PDF) [file pone.0135332.s005.pdf]

**N=5,n=50**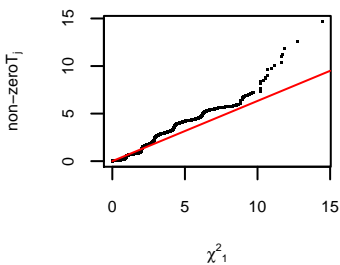**N=5,n=100**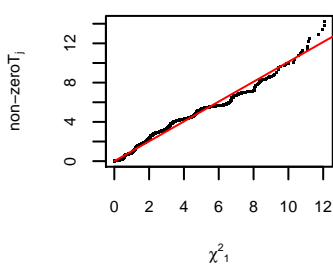**N=10,n=50**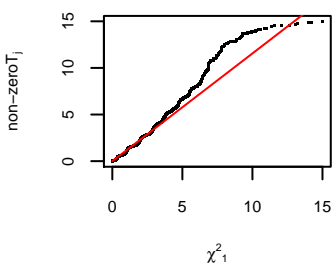**N=10,n=100**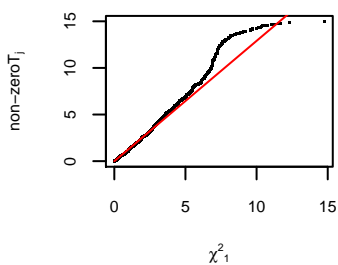**N=20,n=50**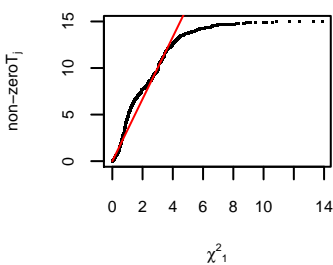**N=20,n=100**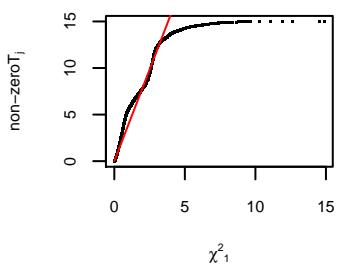

Supplement: S4 Fig — Red straight line has a slope k^. (PDF) [file pone.0135332.s006.pdf]

**N=5,n=50**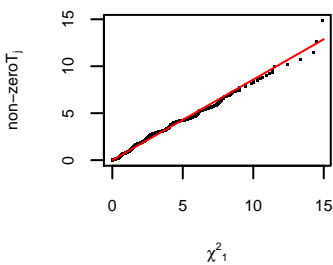**N=5,n=100**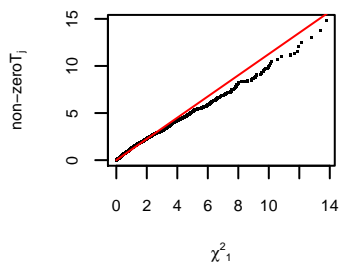**N=10,n=50**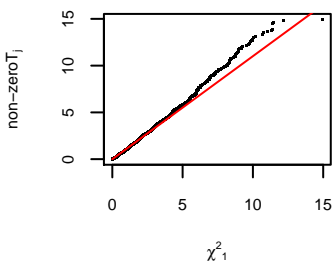**N=10,n=100**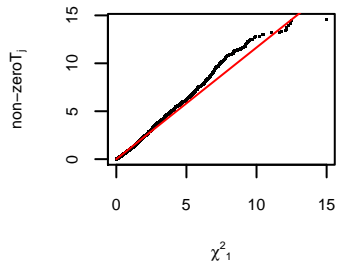**N=20,n=50**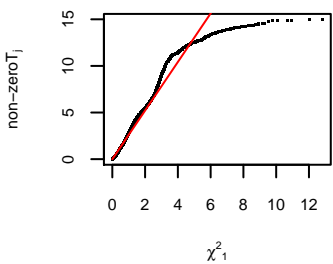**N=20,n=100**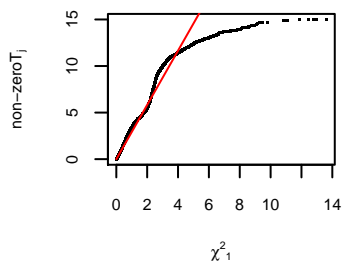

Supplement: S5 Fig — Red straight line has a slope k^. (PDF) [file pone.0135332.s007.pdf]
