# Supplementary material for: MAFsnp: A Multi-Sample Accurate and Flexible SNP Caller Using Next-Generation Sequencing Data
Source: PLoS One. 2015 Aug 26;10(8):e0135332. doi: 10.1371/journal.pone.0135332 (PMC4550471; doi:10.1371/journal.pone.0135332)
Supplement: S1 Table — (PDF) [file pone.0135332.s008.pdf]

N	e	n	$\hat{a}$	$\hat{k}$	N	e	n	$\hat{a}$	$\hat{k}$
5	0.001	50	0.9317	0.3629	20	0.001	50	0.9992	0.7916
		100	0.9357	0.3878			100	0.9991	1.071
		200	0.9421	0.4471			200	0.9987	1.1398
		500	0.9567	0.6675			500	0.9985	1.1079
	0.01	50	0.9336	0.6292		0.01	50	0.9967	1.1677
		100	0.9408	0.9672			100	0.9955	1.2081
		200	0.9347	1.1607			200	0.9947	1.2211
		500	0.9051	0.9768			500	0.9933	1.2204
10	0.001	50	0.9852	0.5095	30	0.001	50	0.9999	0.7392
		100	0.986	0.6517			100	0.9999	0.8317
		200	0.9874	0.9079			200	0.9999	1.2058
		500	0.9877	1.2356			500	0.9999	0.9231
	0.01	50	0.9767	1.0355		0.01	50	0.9996	1.2981
		100	0.9706	1.0945			100	0.9994	1.5058
		200	0.9639	1.0312			200	0.9993	1.3309
		500	0.9587	1.0098			500	0.9991	1.3859
