# Supplementary material for: MAFsnp: A Multi-Sample Accurate and Flexible SNP Caller Using Next-Generation Sequencing Data
Source: PLoS One. 2015 Aug 26;10(8):e0135332. doi: 10.1371/journal.pone.0135332 (PMC4550471; doi:10.1371/journal.pone.0135332)
Supplement: S2 Table — (PDF) [file pone.0135332.s009.pdf]

N	e	n	$\hat{a}$	$\hat{k}$
5	0.001	50	0.9557	0.4352
		100	0.9573	0.5421
	0.005	50	0.9580	0.6331
		100	0.9607	1.0115
	0.01	50	0.9510	0.8580
		100	0.9423	1.1267
10	0.001	50	0.9921	1.7500
		100	0.9906	2.0544
	0.005	50	0.9880	1.1553
		100	0.9834	1.2903
	0.01	50	0.9809	1.1010
		100	0.9751	1.1649
20	0.001	50	0.9970	4.4017
		100	0.9955	4.4773
	0.005	50	0.9979	3.3367
		100	0.9965	3.9323
	0.01	50	0.9974	2.6053
		100	0.9959	2.9126
