# Supplementary material for: MAFsnp: A Multi-Sample Accurate and Flexible SNP Caller Using Next-Generation Sequencing Data
Source: PLoS One. 2015 Aug 26;10(8):e0135332. doi: 10.1371/journal.pone.0135332 (PMC4550471; doi:10.1371/journal.pone.0135332)
Supplement: S3 Table — (PDF) [file pone.0135332.s010.pdf]

N	e	n	SAMtools	GATK	MAQ	seqEM	MAFsnp
5	0.001	50	1.20E-04	0.163	0.006	0.211	0.013
		100	4.20E-05	0.149	0.006	0.178	0.015
	0.005	50	5.00E-05	0.195	0.015	0.437	0.022
		100	1.40E-05	0.187	0.014	0.299	0.008
	0.01	50	9.30E-05	0.290	0.045	0.535	0.009
		100	7.70E-05	0.267	0.044	0.406	0.006
10	0.001	50	9.80E-05	0.160	0.003	0.067	0.011
		100	8.70E-05	0.157	0.003	0.061	0.011
	0.005	50	0.00E+00	0.184	0.002	0.197	0.012
		100	2.50E-05	0.179	0.002	0.138	0.010
	0.01	50	0.00E+00	0.203	0.004	0.280	0.011
		100	0.00E+00	0.197	0.003	0.172	0.011
20	0.001	50	1.30E-05	0.178	0.003	0.041	0.010
		100	4.60E-05	0.174	0.003	0.041	0.010
	0.005	50	0.00E+00	0.182	0.003	0.042	0.010
		100	0.00E+00	0.179	0.003	0.033	0.009
	0.01	50	1.40E-05	0.185	0.002	0.051	0.009
		100	8.10E-06	0.182	0.002	0.038	0.009
Median			1.95E-05	0.182	0.003	0.155	0.010
