# Supplementary material for: MAFsnp: A Multi-Sample Accurate and Flexible SNP Caller Using Next-Generation Sequencing Data
Source: PLoS One. 2015 Aug 26;10(8):e0135332. doi: 10.1371/journal.pone.0135332 (PMC4550471; doi:10.1371/journal.pone.0135332)
Supplement: S4 Table — (PDF) [file pone.0135332.s011.pdf]

N	e	n	SAMtools	GATK	MAQ	seqEM	MAFsnp
5	0.001	50	0.650	0.580	0.710	0.830	0.780
		100	0.610	0.590	0.710	0.830	0.790
	0.005	50	0.510	0.460	0.560	0.810	0.650
		100	0.470	0.440	0.570	0.800	0.610
	0.01	50	0.410	0.260	0.540	0.790	0.530
		100	0.330	0.270	0.550	0.780	0.510
10	0.001	50	0.900	0.900	0.930	0.970	0.910
		100	0.890	0.900	0.930	0.970	0.910
	0.005	50	0.800	0.760	0.850	0.960	0.880
		100	0.760	0.750	0.860	0.950	0.890
	0.01	50	0.700	0.640	0.810	0.940	0.840
		100	0.610	0.640	0.820	0.930	0.850
20	0.001	50	0.990	0.990	0.990	1.000	0.980
		100	0.980	0.990	0.990	1.000	0.990
	0.005	50	0.970	0.970	0.960	1.000	0.980
		100	0.940	0.970	0.970	1.000	0.980
	0.01	50	0.920	0.920	0.940	0.980	0.980
		100	0.790	0.920	0.940	0.990	0.980
Median			0.775	0.755	0.855	0.955	0.885
