# Supplementary material for: MAFsnp: A Multi-Sample Accurate and Flexible SNP Caller Using Next-Generation Sequencing Data
Source: PLoS One. 2015 Aug 26;10(8):e0135332. doi: 10.1371/journal.pone.0135332 (PMC4550471; doi:10.1371/journal.pone.0135332)
Supplement: S5 Table — (PDF) [file pone.0135332.s012.pdf]

N	e	n	SAMtools	GATK	MAQ	seqEM	MAFsnp	Fmax
5	0.001	50	0.790	0.690	0.830	0.810	0.870	0.890
		100	0.760	0.700	0.830	0.830	0.880	0.890
	0.005	50	0.680	0.580	0.720	0.660	0.780	0.820
		100	0.640	0.570	0.720	0.750	0.750	0.820
	0.01	50	0.580	0.380	0.690	0.580	0.690	0.760
		100	0.500	0.390	0.700	0.670	0.680	0.770
10	0.001	50	0.950	0.870	0.960	0.950	0.950	0.960
		100	0.940	0.870	0.960	0.950	0.950	0.970
	0.005	50	0.890	0.780	0.920	0.870	0.930	0.940
		100	0.870	0.790	0.920	0.900	0.940	0.950
	0.01	50	0.830	0.710	0.900	0.820	0.910	0.920
		100	0.760	0.710	0.900	0.880	0.920	0.930
20	0.001	50	1.000	0.900	0.990	0.980	0.990	0.990
		100	0.990	0.900	0.990	0.980	0.990	0.990
	0.005	50	0.980	0.890	0.980	0.980	0.990	0.990
		100	0.970	0.890	0.980	0.980	0.990	0.990
	0.01	50	0.960	0.870	0.970	0.970	0.990	0.990
		100	0.880	0.870	0.970	0.970	0.990	0.990
Median			0.875	0.785	0.920	0.890	0.935	0.945
